# Use it or lose it

**DOI:** 10.7554/eLife.85437

**Published:** 2023-01-24

**Authors:** Ohad Rechnitz, Dori Derdikman

**Affiliations:** 1 Ruth and Bruce Rappaport Faculty of Medicine, Technion – Israel Institute of Technology Haifa Israel; 2 Bnai Zion Medical Center Haifa Israel

**Keywords:** selective vulnerability, entorhinal cortex, chemogenetic silencing, activity-dependent competition, circuit plasticity, Alzheimer's disease, Mouse

## Abstract

Blocking the activity of neurons in a region of the brain involved in memory leads to cell death, which could help explain the spatiotemporal disorientation observed in Alzheimer’s disease.

**Related research article** Zhao R, Grunke SD, Wood CA, Perez GA, Comstock M, Li MH, Singh AK, Park KW, Jankowsky JL. 2022. Activity disruption causes degeneration of entorhinal neurons in a mouse model of Alzheimer’s circuit dysfunction. *eLife* **11**:e83813. doi: 10.7554/eLife.83813.

Alzheimer’s disease is a neurodegenerative disorder that affects approximately 55 million people globally (Gauthier et al., 2021). One serious symptom of the disease is a tendency to become disorientated both in space and in time. Researchers attribute this, in part, to impairments in the medial temporal lobe of the brain, which includes the hippocampus and the entorhinal cortex. These two regions are involved in the formation and retrieval of memories; in particular, they are important for making spatial memories for navigation, including memories of place, time, head direction and environmental borders (see for example review by Knierim, 2015).

To encode new memories and retrieve past ones, neurons in the network formed by the entorhinal cortex and the hippocampus have to maintain their plasticity – that is, their ability to form new connections between cells and remove ones which are rendered obsolete **–** throughout life. In mouse models of Alzheimer’s disease, however, the role of hippocampal and entorhinal cells in learning and memory is impaired (Rechnitz et al., 2021; Ying et al., 2022). As these regions are highly interconnected, early-stage corruption in the entorhinal region is further amplified downstream in the hippocampus. This leads to a vicious cycle that may impact behavior and cause spatial and temporal disorientation.

The specific mechanisms leading to a deterioration of the brain’s neural networks in Alzheimer’s disease are unclear. Similarly, it is unknown how this leads to memory loss. However, the impairment of neural networks within the entorhinal cortex often appears early in the progression of the disease (Stranahan and Mattson, 2010). Now, in eLife, Joanna Jankowsky and colleagues at Baylor College – including Rong Zhao, Stacy Grunke and Caleb Wood as joint first authors – report on how a neuronal population in layer II of the entorhinal cortex contributes to this impairment (Zhao et al., 2022). Their experiments show that blocking the activity of these neurons ultimately leads to increased cell death in this population.

Zhao et al. used a mouse model thought to undergo the same disruption to cell activity observed in Alzheimer’s disease. However, in this model, the impairment is not induced by the amyloid-beta plaques or neurofibrillary tangles characteristic of the disease. This is interesting because, even though it had previously been established that neurons die during Alzheimer’s disease, this was usually attributed to a vicious cycle of increased amyloid release driven by increased synchronization between cells causing hyperactivity (Busche and Konnerth, 2015; Zott et al., 2019). Instead, Zhao et al. show that, in their mouse model, cell deterioration and death in the entorhinal cortex are driven by silencing of specific neuronal activity.

Zhao et al. also demonstrate that, in their mouse model, a competitive process between active and inactive cells in the entorhinal cortex precedes neurodegeneration. This type of competition is similar to what is seen in infantile neuronal plasticity during development, when neural circuits are refined by selecting neurons and neural pathways depending on their levels of activity. Following this refinement process, which was previously thought to end soon after birth, some neurons degenerate while others persist. The extension of infantile neuronal plasticity in entorhinal cortex cells into adulthood may act as a double-edged sword: on the one hand, the plasticity allows these cells to form and modulate memories throughout life; on the other hand, the cells are more vulnerable to malfunction and death through competition.

Sensory information from multiple modalities (i.e. tactile, olfactory, auditory etc.) converges into layers II and III of the entorhinal cortex, which form the major input sources for two regions in the hippocampus, called the dentate gyrus and CA3. These areas of the brain are part of a pathway that ultimately terminates in another hippocampal region known as CA1 (Witter et al., 2000). It is therefore suggested that the cell impairment observed in the entorhinal cortex by Zhao et al. resembles an isolated deficit that can occur in Alzheimer’s disease. This impairment potentially forms an early seed to the later deterioration of neural networks and brain regions downstream, mainly in the hippocampus (Figure 1; Cacucci et al., 2008; Rechnitz et al., 2021; Roy et al., 2016). This may lead to the memory deficits and disorientation observed in Alzheimer’s patients.

**Figure 1.**
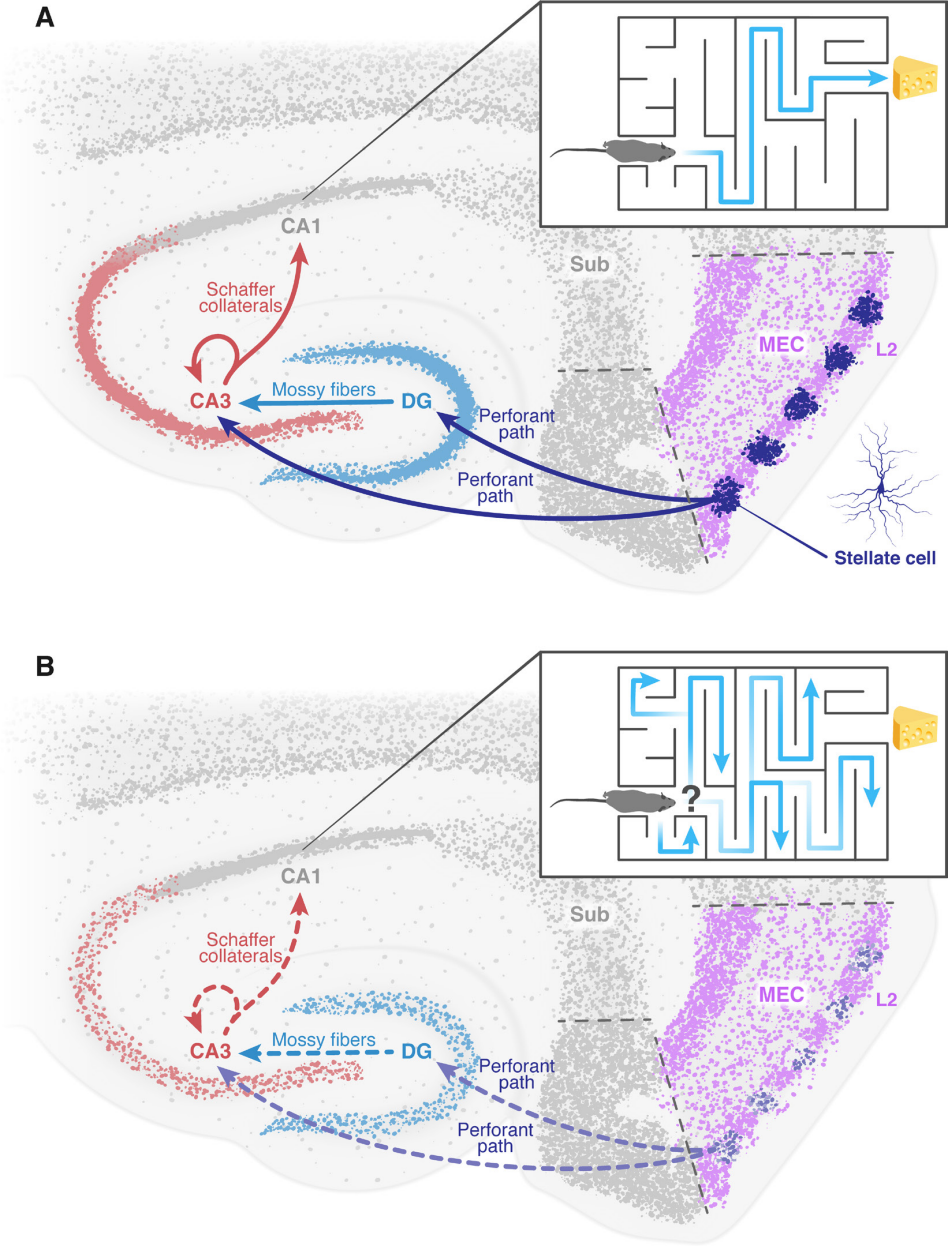
The deterioration of neural networks following impairment of the entorhinal cortex may drive disorientation in Alzheimer’s disease. (**A**) In healthy mice, the tri-synaptic pathway begins in the entorhinal cortex (MEC, pink) and terminates in the CA1 region (grey) of the hippocampus. A prominent population of stellate cells (dark blue) can be found in layer 2 (L2, pink) of the entorhinal cortex. First, signals travel from layer 2 of the entorhinal cortex through the perforant path (dark blue arrow) to the dentate gyrus (DG, light blue) and the CA3 (red). Signals then travel from the dentate gyrus to the CA3 through the mossy fibers (light blue arrow). From the CA3, signals are transmitted through axons known as Schaffer collaterals (red arrows) to the CA1. This pathway is known to be essential for the formation of spatial memory and navigation. The inset shows how a healthy mouse, in which this pathway is working correctly, can navigate a maze. (**B**) In mice models of Alzheimer’s disease, the results of Zhao et al. suggest that the stellate cells in the entorhinal cortex degenerate and corrupt the information transmitted through the tri-synaptic pathway (dashed arrows), leading to the disruption of the neural network downstream, neuronal loss and ultimately disorientation. The inset shows how an Alzheimer’s disease model mouse is unable to navigate a maze. MEC: medial entorhinal cortex; Sub: subiculum.

Figure credit: Dafna Antes.

The findings of Zhao et al. hold promise for potential new treatment strategies targeting the cell population in layer II of the entorhinal cortex early on in the Alzheimer's disease. These interventions have the potential to slow down the cascade of events leading to the onset of the condition.
